# Parents’ Experiences with Online Screening Tools in Well-Child Clinics and School Health Services: A Qualitative Study

**DOI:** 10.1177/23333936251342728

**Published:** 2025-06-10

**Authors:** Trine Holm, Elin Thygesen, Geir Inge Hausvik, Thomas Westergren

**Affiliations:** 1 University of Agder, Grimstad, Norway; 2 University of Stavanger, Norway

**Keywords:** technology, parenting, communication, nursing, pediatric, children, growth and development, Norway

## Abstract

The aim of this study was to explore the experience of parents who completed online screening tools about their child’s health, development, and well-being, and parents’ experiences with the public health nurse’s handling of this information during a well-child visit for children aged 0 to 7 years. Twenty well-child visits were observed, and 16 parents were interviewed individually or in pairs using a semi-structured interview guide. The parents’ experiences were explored using reflexive thematic analyses of verbatim transcripts and field notes. Five main themes were developed; *Experiencing Ease of Use and Confusion*, *Evoking Novel Insights, Evoking Insecurity and Vulnerability, Evoking New Expectations* and lastly, *Navigating Expectations*, consisting of two sub themes; *Harnessing Potentials* and *Neglecting Potentials.* These findings indicate that online screening tools might provide important benefits, provided that parental insecurity and vulnerability are addressed by information and dialogue.

## Background

In Norway, all families are offered free regular appointments for their children from birth to age 16 years at well-child clinics and/or school health services. The services are led by public health nurses (PHNs) and aim to promote and protect the healthy development of children and identify early deviations in physical and social-emotional development. During the early years, the services also aim to increase parents’ health literacy and facilitate positive interactions between parents and children (The Norwegian Directorate of Health, 2017).

Although these services are well developed, there is considerable variation in how they are delivered. World Health Organization designed screening criteria to ensure the tools are easy to use, and the outcome is interpretable, acceptable, accurate, reliable, sensitive, and specific (Wilson & Junger, 1968). A recent review of national practices in the Nordic countries and Scotland revealed that none of these countries fulfilled these criteria (Wilson et al., 2018). The variation of use is partly because of the reliance on experience-based assessments. Structured parent- or child-reported tools and tracking of a child’s health, development, and well-being are used infrequently except to document physical growth. For this reason, such information is not recorded in a structured format in electronic patient records (Wilson et al., 2018). To address such gaps, valid and reliable tools for the early identification and evaluation of a child’s development are recommended to be integrated into routine child health services (Alexander et al., 2017; Shonkoff et al., 2021). In this article we use the term “screening tools” as it consists of validated questionnaires. However, we emphasize that such tools in this context are not used for diagnostics but to elicit information and awareness about health challenges as well as to facilitate dialogue and health promotion as intended in primary child health services (Garner et al., 2021; The Norwegian Directorate of Health, 2017).

The importance of assessing a child’s health reflects the findings that unhealthy trajectories might start early during childhood and that early experiences provide the base for brain development and general functioning throughout life (Boyce et al., 2021; Shonkoff et al., 2021). Thus, the early childhood period is considered the optimal time for identifying and addressing early signs of developmental delays before they develop into permanent patterns (Poulou, 2015). Investing in early interventions for improved child health benefits children at risk and has substantial societal returns (Heckman, 2008). These interventions should be grounded in evidence-based practices, which necessitate a thorough assessment of the potential concern before implementing interventions (Alexander et al., 2017). However, implementing screening tools into these services presents challenges. The tools must be usable, acceptable, and affordable for users and facilitate understanding, dialogue, and engagement concerning challenges without labeling the child or the family (Shonkoff et al., 2021). However, the lack of integration into PHNs’ workflow and the additional workload for PHNs are barriers to the widespread use of such tools (Sheeran et al., 2021). Online solutions and the parent’s responses to the screening tools before appointments are preferable and more acceptable for PHNs because these reduce the cost and completion time and increase the response rate and accuracy (Bodnarchuk & Eaton, 2004; Guralnick, 2017).

During early childhood, parents play an important role in supporting their child’s development. The parents’ concerns are often the first sign of developmental challenges and thus merit significant weight in the evaluation process (Marks et al., 2011). However, previous research has reported barriers between parents and PHNs in addressing challenges that affect children by hindering early identification and preventive interventions (Gellasch, 2016). Parents report barriers such as lack of knowledge about normal development, stigma, labeling, and fear of being viewed as bad parents (Brinley et al., 2024; Traube et al., 2021). When raising concerns, parents often think that PHNs are inattentive and sometimes provide false reassurance (Gellasch, 2016). At the same time, PHNs fear that raising concerns despite uncertainty might harm the parent–PHN relationship and create unnecessary worry or anxiety for parents (Gellasch, 2016). These situations might lead to missed opportunities for discussing a child’s development and initiating preventive measures, which can cause developmental issues to go undetected in some children who would benefit from early intervention (Hix-Small et al., 2007; Marks et al., 2009). Therefore, it is important to find solutions that help parents feel more comfortable communicating their concerns to PHNs.

In addition to identifying more at-risk children compared with standard care (Hix-Small et al., 2007; Marks et al., 2011), the implementation of screening tools into services might facilitate user involvement, initiate better communication between parents and PHNs, and improve service quality (Bagner et al., 2012; Eklund et al., 2009; Fothergill et al., 2013; van Minde et al., 2018; Waldron et al., 2018). Furthermore, parents perceive completing screening tools as acceptable (Valla et al., 2019). However, the use of such tools should not come at the expense of dialogue and attentively listening to parents’ concerns (Borg et al., 2014; Valla et al., 2019).

Previous research has shown that parent–PHN communication, parent trust in PHNs, and comfort in talking with PHNs are needed to induce parents to follow through with PHN recommendations (Fuzzell et al., 2018; Mărginean et al., 2017). Thus, understanding parents’ experiences with the use of screening tools is essential for several reasons. First, such tools can influence both the accuracy of the information provided and the parent’s level of engagement in the process. Second, studying these experiences might provide valuable insights into how the implementation of online screening tools influences the known barrier of addressing children’s challenges during well-child visits. Third, gathering this information is crucial for driving practical changes in service delivery by ensuring that these tools are used in ways that effectively meet parents’ needs and support optimal outcomes for children.

In the present study, we examined parents’ experiences in completing online screening tools and their experiences of the PHNs’ use of the information obtained from these tools. We asked the following research questions: How do parents experience their completion of online screening tools about their child’s health, development, and well-being?; and How do parents perceive the PHN’s handling of the information obtained in well-child visits for children aged 0 to 7 years?

## Methods

### Context

This study was conducted within the larger *Starting Right* project, which is a research-based innovation established to streamline and improve the service processes for children (Westergren et al., 2021). This project provides a population overview and identifies children who have or are at risk of developing poor health and reduced quality of life. In this project, an online child- or parent-reported screening tool was implemented to collect health data for children and facilitate dialogue between the parents, child, and PHNs within well-child clinics and school health services. When this study was undertaken, eight municipalities in southern Norway participated in *Starting Right*.

Within Starting Right, validated parent-reported questionnaires were used to assess various aspects of child development and well-being. General mental health was measured using the Strengths and Difficulties Questionnaire (SDQ), consisting of 25 questions with response alternatives “not true,” “somewhat true,” or “certainly true” (Sveen et al., 2013). Health-related quality of life was measured with KIDSCREEN-27, which includes 27 questions answered on a 5-point Likert scale indicating frequency or intensity of a behavior or feeling (Andersen et al., 2016; Ravens-Sieberer et al., 2007). General development was measured using the Ages and Stages Questionnaire (ASQ), with 30 questions, representing five domains with six question each, and response alternatives “yes,” “sometimes,” or “not yet” (Rasmussen & Martinussen, 2013). Social-emotional development was measured using the Ages and Stages Questionnaire: Social-Emotional (ASQ-SE), containing 19 to 33 questions with response alternatives “often or always,” “sometimes,” or “rarely or never” (Stensen et al., 2018). For ASQ and ASQ-SE, there is also a separate column where parents can indicate if their child’s behavior causes them concern. The *Starting Right* project facilitated the distribution of questionnaires at 6 months, as well as at 2, 4, and 6 years of age. However, each municipality had the flexibility to determine which questionnaires to implement and for which age group. The screening tools were distributed to both parents before the well-child visit through a text message using a validated high-security individual Internet link and secure identification through the Norwegian public e-services login system (ID-porten). Parents who did not complete the online screening tool within 1 week after receiving the link received a reminder text message. Parents of children aged 0 to 7 years completed the online screening tool before well-child visit with a PHN at the well-child clinic or school health service. Before the scheduled well-child visit, PHNs could access a summarized report on the child generated from each questionnaire, including cut-off scores indicating whether the child’s results were within the normal range or not. The *Starting Right* project also included self-reported questionnaires for children from age 8 years within the school health context; however, these were not explored in the present study.

### Study Design

This qualitative study, consisting of observations and interviews, is grounded in the common ontological and epistemological grounds of phenomenology, hermeneutics, and existentialism as described by Dahlberg and Dahlberg (2020). Our approach emphasizes the concept of inseparability, meaning that “experience is born from the world, directed to the world, and must be understood with the world as a background” (Dahlberg & Dahlberg, 2020, p. 460). From this perspective, knowledge is developed in closeness to the field and are both descriptive and interpretative, while trustworthiness is ensured through reflexivity. Moreover, rather than being a choice of either-or, the relation between subjectivity and objectivity comes into attention and investigation shaped through the empirical material and the researcher’s interpretation of participants’ experiences, influenced by their own assumptions, values, theory, and context. The research team consists of researchers from different disciplines, including nursing science (TH, ET, and TW) and information systems science (GIH). Two of them were connected to an e-health research center, and TW was the initiator and previous project leader for the project *Starting Right*. TH, who was responsible for data collection, is in her thirties and has children in the same age group as the participants. Although TH had no personal experience with the online screening tool, as her child’s follow-up care was not part of the program, she was invited to the Starting Right project meetings. Authors’ experiences shaped our situatedness as researchers and our lens for analysis. The study employed an inductive approach, with the analysis “grounded in the data” (Braun & Clarke, 2022). Methodological integrity was ensured by aligning the research design, research questions, and theoretical assumptions. The reporting followed the Standards for Reporting Qualitative Research (O’Brien et al., 2014).

### Participants and Recruitment

To recruit PHNs, TH contacted the leaders of the well-child clinics and school health services incorporated in Starting Right. The leaders distributed information about the study to PHNs along with contact information to TH. The PHNs who agreed to participate then contacted TH for additional information and scheduled when data collection could take place. Seven PHNs within three municipalities associated with five well-child clinics and two schools agreed to participate. Before the scheduled well-child visits, PHNs contacted the parents via phone or text to provide information about the study and obtain verbal consent for the presence of TH during the well-child visits. At the start of the well-child visit, TH provided additional information about the study to the parents. Parents who also expressed interest in being interviewed provided contact information.

A total of 20 well-child visit observations were conducted, and 19 parents provided contact information. Seven of these parents were not included, either because TH was unable to establish contact with them after the observation, or because of concerns about recall bias resulting from the long interval between the observation and the interview opportunity. In some cases, only one parent attended the well-child visit with the child, although both parents were involved in completing the online screening tool before the well-child visit. In these cases, the other parent was invited to participate in the interview with the partner to gather his or her perspectives about completing the online screening tool. Since the six-year health consultation follows the child’s first school year rather than the child’s age, some children included in the study had already turned 7 by the time they attended the health consultation. Table 1 provides an overview of participants involved in data collection. The characteristics of the participants can be found in Table 1. However, characteristics were only collected from parents who agreed to be interviewed, and thus, only their characteristics are included in the table.

**Table 1.** Participants Characteristics.

Characteristics	Parents observed (*n* = 23)	Parents interviewed (*n* = 16)	PHNs observed (*n* = 7)
Women (*n*)	18	11	7
Men	5	5	—
Age (years)	—	34 (27, 53)	50 (27, 62)
Number of children in the family	—	2.5 (1, 4)	—
*Childs gender*			
Boys	10	5	
Girls	10	7	
Age of child	6 (0, 7)	5 (0, 7)	—
In person interviews	—	4 (*n* = 12)	—
Remote interviews (zoom or telephone)		8 (*n* = 12)	
*Education of parent*			
High school (*n*)	—	5	—
Bachelor’s degree (*n*)	—	5	—
Master’s degree or higher (*n*)	—	5	—
Not accounted for	—	1	—
*Experience with questionnaire*			
SDQ (*n*)	14	9	3
KIDSCREEN (*n*)	9	6	3
ASQ-SE (*n*)	6	7	5
ASQ (*n*)	8	7	5

*Note*. Numbers are given as median (min, max) unless otherwise stated. PHN = public health nurse; ASQ = Ages and Stages Questionnaire; ASQ-SE = Ages and Stages Questionnaire, Social–Emotional; SDQ = Strength and Difficulties Questionnaire.

### Ethical Considerations

Ethical approval was given by the Norwegian Centre for Research Data (Number: 387959). All participants gave their written informed consent and were informed that all information would be treated confidentially. All parents were informed that participation would not have any impact on the care from the well-child clinics or school health services. However, it is important to acknowledge that the presence of TH during the well-child visits might have influenced those specific interactions.

### Data Collection

Observations and interviews were conducted between May and December 2022.

#### Observations

The purpose of the observations was to gain insight into how the information obtained from the screening tool was handled by the PHN in the well-child visit between the PHN, child, and parents. During the well-child visits, keywords were documented and expanded into more detailed notes shortly thereafter. These field observation notes were then sorted as methods, descriptive data, and reflective notes (Hammersley & Atkinson, 2007). The participants’ names were replaced with codes to ensure confidentiality. A list linking the names and codes was stored safely and separately from the dataset.

#### Interviews

The interviews lasted 26 to 70 min and were audio recorded and conducted within 2 weeks after the well-child visit. The interviews were held either in person at the parents’ home (*n* = 4), via Zoom (*n* = 5), or over the phone (*n* = 1), according to the parents’ preference. In four cases, both parents were interviewed together. When interviews were held via Zoom or telephone, anonymity and confidentiality were ensured by sending a private invitation to the participants, who had to log on with a password and be admitted by the host. The researcher conducted the interview alone in a room to ensure no one could attend or overhear what was said.

A semi-structured interview guide (Supplementary File 1) was used to ensure that all participants shared their experiences with the following topics: completing the online screening tool, the tool’s content, interactions with the PHN after completing the tool, and their thoughts on how such screening tool could be used most effectively. The information obtained during the observation of the well-child visit was used to remind the parents of the topics discussed. Follow-up questions were also asked to obtain supplementary reflections from the parents. All interviews were transcribed and aligned with the observation notes; the participants’ names were replaced with a code, and any other identifying information was changed. For example, if participants named their child in the interview, the name was marked as [name of the child].

### Analysis

Data were analyzed using reflexive thematic analysis, following the six-phase approach outlined by Braun and Clarke (2022), with a focus on semantic-level interpretation. This approach promotes a dynamic and interactive approach between the phases. TH led the analytical process, enabling a reflective approach to the data. The co-authors contributed valuable insights and reflections by reviewing the coding process, the thematic structure, and drafts of the findings. Together, they assessed the analytical results by posing critical questions to ensure a robust and thorough analysis.

Familiarization with the data began during the data collection, as TH took reflective notes following each interview and observation. This continued through the transcription process and multiple readings of the material prior to systematic coding. During this phase, TH also took notes of potential patterns and preliminary themes. All interview and observational data were organized and coded using NVivo11 (2015). Initially, coding was conducted using language directly derived from the participants to maintain closeness to the original data. Subsequently, codes were reviewed, refined, and merged where appropriate. The themes were identified based on the semantic content of the data, emphasizing participants’ expressed experiences and perspectives. These themes were iteratively reviewed to ensure internal coherence and consistency across the dataset. Once finalized, TH revisited the entire dataset to confirm that the themes accurately reflected the data as a whole. Finally, each theme was clearly defined and named to encapsulate its central meaning, culminating in the writing up of the analytical findings and the overall report (Braun & Clarke, 2022). Quotations from the participants have been translated from Norwegian to English with support from a native English-speaking editor.

## Findings

Parents’ experiences are covered and presented in the following five main themes; Experiencing Ease of Use and Confusion, Evoking Novel Insights, Evoking Insecurity and Vulnerability, Evoking New Expectations and lastly, Navigating Expectations, consisting of two sub themes; Harnessing Potentials and Neglecting Potentials.

### Experiencing Ease of Use and Confusion

This theme presents the variation found in usability with the digital solution and how it was to complete the online screening tool. Most parents found it advantageous that the screening tool was online and sent out in advance of the consultation, which made it easy to access and complete it at their convenience via a link they had received by phone. They noted that the number of questions was manageable, that it only took a short time to answer them, and that it was easy to navigate within the screening tool. A mother of a 6-month-old boy explained:

It was actually really nice. I just used my phone. It wasn’t like it jumped around or was too big or anything. It was really, yeah, actually really straightforward. And then I just had to press next, and it took me to the next page. (P114)

However, some parents reported some confusion about the presence of multiple questionnaires. Some were unaware that they had not completed all the questions until they received a reminder, and others became aware of this only during the well-child visits. A mother of a seven-year-old boy explained:

I received feedback from the well-child clinic that I had only answered the first questionnaire and didn’t go any further. [That was because] when I was answering these questions, it suddenly jumped back to the first page. And then I thought I was done. (P105)

Additionally, a few parents found it difficult to understand the questions because of factors such as dyslexia or Norwegian not being their first language. These challenges led to misunderstandings that were addressed later during the well-child visit. A father of a two-year-old girl explained, “So, it has been a problem over the years [having dyslexia]. (. . .) I think it’s a bit tricky when you get those kinds of questions thrown at you just like that. I know I’ve answered some of them incorrectly.” (P119)

### Evoking Novel Insight

This theme addresses the positive feelings parents experienced while responding to the online screening tool. They found the content of the screening tools important and relevant and had positive outcomes for them as parents. Parents reported gaining new and deeper insights into their child’s physical and psychological development through the process of completing the screening tool. Those who completed the ASQ questionnaire about their child’s physical development felt that they now had a better understanding of their child’s capabilities. This newfound awareness influenced how they viewed and interacted with their child, which helped to make activities and playtime more enjoyable. A father of a 6-month-old girl described the outcome of learning new insights as follows: “It has actually become a bit more fun to play with her or do stuff with her after we became more aware that she can do more things than we previously realized.” (P113)

In terms of their child’s mental well-being, parents noted that they became more reflective and mindful of their child’s emotional state and more aware of the factors influencing their child’s daily life. A mother of a six-year-old boy explained this shift as follows:

I started thinking a bit more about how he’s doing. More consciously—it’s kind of an awareness thing. There are a lot of questions here and a lot of elements, like, um. . . yeah. It sort of encompasses all of him. So, in that sense, it’s really good to get a reality check on what can affect him in everyday life. (P123)

### Evoking Insecurity and Vulnerability

This theme describes how parents experienced insecurity and vulnerability when responding to the online screening tool. The uncertainty began as soon as they received the online screening tool, even before they started answering them. A mother of a seven-year-old explained that she felt unsure about the purpose of the screening tool: “I wasn’t entirely sure what was intended. Was it primarily for the well-child clinic or mainly for research purposes, or like, what was the main objective, in a way.” (P101) During the completion of the screening, even more uncertainties arose. They wanted to give an accurate picture of their child; however, they became uncertain about how to do so. For example, when answering questions about their child’s inner feelings, as asked within KIDSCREEN, parents described it as challenging because the child chose what they wanted to share with their parents. This was exemplified by a mother of a six-year-old girl: “It’s a bit tricky to know if she feels lonely because she only shares what she wants to when she gets home. She might not want to share all her impressions from school either.” (P125)

Parents also experienced uncertainty about what constituted normal or abnormal development and feelings and reported difficulties choosing from limited response alternatives. They believed that children expressed a broad spectrum of emotions and behaviors, and that more nuanced response alternatives or the ability to add comments were needed. This is illustrated by a statement from the mother of a seven-year-old girl:

Completing tasks, good concentration. Not true, somewhat true, certainly true. But then, um, you often want to write something extra. (. . .) Because sometimes she’s [the daughter] super focused on tasks and stuff, and other times she can’t concentrate at all, which I think is completely normal for all kids. (P102)

Several parents also expressed uncertainty regarding the intentions behind the screening and the PHNs’ handling of their responses, and reported feeling vulnerable when completing the screening tool, which evoked a range of concerns. Parents feared being perceived as inadequate, got concerned about stigmatization, and unnecessary evaluations that could negatively impact their children and families. One mother described it as follows: “You’re making yourself quite vulnerable, opening up about, essentially, your entire life and existence.” (P123) This vulnerability extends even to those without immediate concerns, because of the fear of potential misinterpretation and its consequences. For example, a mother expressed her hesitation, wondering if inaccurately depicting her daughter’s restlessness, because of to her high energy, might prompt unnecessary actions, such as a comprehensive ADHD assessment: “If I said she couldn’t do it at all, would the well-child clinic then initiate an extensive assessment and ADHD evaluation based on that answer?” (P125) This uncertainty could make honest responses challenging and was particularly acute for parents with prior experience of being under investigation for being fit as a parent. Past experiences could influence their willingness to be forthcoming. As a mother of a seven-year-old boy shared;

I might not want to write that my child has difficulties (. . .) Maybe I feel like I should have been able to get him to be a bit more obedient, you know, for me. It might have been easier for me to bring up that kind of stuff if we hadn’t gone through that concern. (P101)

### Evoking New Expectations

When online screening tools were implemented, parents described new expectations regarding the follow-up they would receive at the well-child visits. Although they were unsure how these tools would be used, they formed their own opinions about the benefits such tools could offer and, consequently, how they should be approached in the well-child visits.

Parents expected the PHN to review their responses before the well-child visit so that both they and the PHN were better prepared for the appointment. This preparation was expected to manifest using results during well-child visits, either by presenting them in graph form or by asking more targeted questions, addressing the family’s positive aspects and potential challenges. As one parent remarked, “It might be easier for the public health nurse to ask targeted questions if you’ve filled that out. Because then they can get to something she might not have had time to ask about during the consultation.” (P101)

Parents meeting to well-child visits with children 4 to 7 years also expected direct engagement with their children on the topics from the screening tool, underscoring the significance of involving the child’s perspective. As a mother of a seven-year-old girl noted, “I think it’s important that they ask the kids how they’re doing. That they are allowed to answer too, especially when they get older. Because they do. Yeah. They do answer.” (P102)

By using the screening tool in this way, parents expected the conversation and discussion to result in support and guidance on how to handle any concerns they had about their child, to prevent them from escalating or becoming long-term issues. Most parents noted that they found it sufficient to receive guidance from the PHN on how they could support their child during the current phase. However, when facing major challenges, parents expected that PHNs would utilize information from online screening tools to conduct comprehensive assessments and possibly initiate referrals to specialists.

### Navigating Expectations

In practice, interviews and observations showed notable discrepancies in how PHNs incorporated screening information into well-child visits. Some PHNs employed the results as a framework for discussion, visually presenting data and exploring specific areas further with parents or children, meeting parents’ expectations. In contrast, other PHNs observed mentioned the results only in passing or omitting them entirely from the consultation, choosing instead to follow national guidelines independently of the screening tools insights. This main theme consists of two sub-themes exploring the variation in the PHNs handling of the information in the well-child visits and how this was experienced by the parents.

#### Harnessing Potentials

This sub-theme highlights the potential benefits of using online screening tools when the information from these was discussed during a well-child visit. Observations revealed several instances where PHNs engagingly integrated the screening results into discussions. For example, one PHN discussed a child’s drawing abilities before engaging them in related activities, using the screening responses as a starting point for interaction with the child. Another PHN started the conversation by addressing the screening responses, showing the mother a graph of results, explaining the child’s strengths and challenges, and engaged both the child and the mother in the conversation.

When PHNs actively integrated the screening responses into the conversation, as in the examples, parents found it beneficial. They felt that completing the screening tool allowed for a more direct address of challenges, facilitating a structured conversation and leading the well-child visits to be more equal for all. This was described by the mother of a two-year-old as:

The form gives a little. . . it provides a chance for conversation about certain things. I think. . . and it might feel a bit more consistent no matter who your public health nurse is. They have a tool to cover everything, you know? (P116)

These discussions often evolved into collaborative dialogues where the PHN, children, and parents together discovered what was working well and what improvements could be made. When the screening responses were used in such a dynamic way, parents reported feeling secure and greatly valued the guidance, whether it pertained to handling emotional challenges or tips to develop specific skills. For a family that previously was struggling to get help, it was a great relief to feel that their challenges were now taken seriously. They believed that the early help they were receiving was a result of the PHNs’ trust in standardized tools rather than just parental concerns. The father noted:

It was easier to identify certain problem areas because when you have a form, you can quantify it more easily for professionals regarding what’s frustrating. Meanwhile, when you come there as a new father—or very often a mother—with the worries, I assume the public health nurse needs to filter a bit. (P109)

#### Neglecting Potentials

This sub-theme explores how the potential benefits of using screening tools in well-child visits were neglected when results were not thoroughly discussed and integrated into conversations. Parents who experienced little or no discussion of the screening responses described the topics discussed as random, depending on the assigned nurse, and it was up to the parents to highlight their perceived challenges. Our observations supported this experience, indicating that PHNs often followed guidelines from Norwegian health authorities but did not necessarily cover all topics in every consultation, leading some topics to be unaddressed. PHNs often asked parents if they had any concerns or topics they wanted to address and then focused on these. Sometimes, parents responded that they could not think of anything or that everything was fine, even though later in the conversation, they touched on areas they found challenging. A mother of a six-year-old explained:

Because it’s a bit random what gets brought up and when we’re asked that question [if there’s anything else we want to say] . . . once you’ve filled out such a comprehensive questionnaire, I think it might be good to address the things that score quite high. . . So, it shouldn’t feel like now I have to tell them how difficult things are, or something like that. It’s not always easy to remember everything either. (P123)

Parents who had completed the screening tool beforehand often hesitated to discuss the challenges again, assuming they were deemed normal by the PHNs if not mentioned, even though the parents themselves felt the need for guidance. As a mother of a seven-year-old boy noted:

So, she [the PHN] might have picked the things she thought were important to address. And other things that are probably normal for that age, like not always telling the truth or maybe trying to take things that aren’t theirs. Those are things you need to address, but she might not think they’re super important because that’s just what 6-year-olds do. (. . .) But it’s harder to tell again if I’ve already answered that he does this. (P101)

The mismatch between expectations and actual use caused some parents to question the value of the screening tool. A mother of a 6-month-old girl expressed frustration, stating:

It’s just something we have to do. We take a look at it, but I guess if I had scored really high in some areas, we would have spent a bit more time on that, maybe. I hope so, at least, because otherwise, I believe, like, the whole form is wasted, so to speak. (P115)

## Discussion

This study provides insights into parents’ experiences completing an online screening tool regarding their children’s health, development, and well-being before attending a well-child visit with a PHN, and the parents’ perceptions of how PHNs handle the information obtained. The findings not only highlight several benefits of implementing such tools, but also illustrate the parents’ concerns, expectations and needs in relation to their use of the services.

Overall, the parents in our study reported high accessibility and usability of the online screening tool. They appreciated that it was possible to complete online, at their convenience, and with ample time to reflect on and observe their children, and that this helped to ensure accurate responses. Previous research has identified various barriers to responding to online screening tools, including language difficulties (Sheeran et al., 2021), security concerns, technical issues, and a lack of digital skills (Meirte et al., 2020). In the present study, misunderstandings because of language difficulties were mentioned. However, no mentions were made of security concerns or a lack of digital skills, although it is likely that parents with security concerns or a lack of digital skills would not have completed the screening tool and thus not been recruited. Children from culturally and linguistically diverse backgrounds and low-income families often have lower uptake rates of developmental programs (Ayer et al., 2020; Woolfenden et al., 2015) despite being at a higher risk for developmental delays and mental health issues. These children seem to benefit most from early identification and intervention (Larson & Halfon, 2010).

Parents did not always complete all the information requested by the PHN. This misunderstanding arises because of the online format of the screening tool, which may lead to incomplete responses and, consequently, certain topics not being addressed during the well-child visit. Possible strategies to address this challenge include incorporating a feature to display the respondent’s progress through each questionnaire and/or providing feedback confirming the form has been successfully completed and submitted (Couper et al., 2013).

The themes, *evoking insight* and *evoking insecurity and vulnerability* showed that parents were ambivalent about completing the online screening tool. Consistent with previous research, parents expressed positive attitudes about the themes that were discussed. They also reported that they, by responding to the online screening tool, became more reflective and aware of what was expected of their child at various stages and were therefore better prepared for the well-child visit (Brinley et al., 2024; Nelson et al., 2011; Traube et al., 2021). However, aligned with parents’ experiences, when a provider administered the screening tools (Brinley et al., 2024), feelings of vulnerability and insecurity during the screening process were mentioned. These perceptions stemmed from their worries about labeling and potential negative consequences for the child and their family, as well as concerns about whether their child was developing normally. This, in turn, made it crucial to provide an accurate picture of their child, which was challenging given the narrow response alternatives. It also prompted them to reflect on what was considered normal development, behavior, and emotions for their child’s age.

The early childhood period is characterized by rapid changes in both physical and emotional development, and the range for what is “normal” is wide (National Research Council and Institute of Medicine, 2000). However, even if some children outgrow negative behaviors or emotions, or catch up with their physical development, previous research has shown that problems early in life tend to persist or escalate if not handled (Poulou, 2015). Therefore, it is important to ensure that parents are comfortable reporting honestly and accurately about their child’s health, development, and well-being. To address these negative emotions and ensure parent comfort, Brinley et al. (2024) suggested that information and reassurance throughout the screening process could be beneficial. The parents in our study were not asked directly about the information they received before completing the screening tool. However, several participants mentioned that they were unsure about the purpose of the screening and how the results would be used. These perceptions indicate that additional clarification is needed to reassure parents and promote their honest reporting.

The way in which PHNs discuss the results of the screening tool during well-child visits can greatly impact parents’ feelings of vulnerability. Variations in how the online screening tools were used during the well-child visit appeared to be linked to parent satisfaction with the PHNs handling of the information. Negative emotions persisted when the results were not discussed during well-child visits. This made it more difficult to address parents’ concerns about their child’s health, development, or well-being, as some parents even hesitated to bring up concerns reported in the screening if the PHN did not mention them. It is therefore important not only to implement an online screening tool into practice in well-child clinics and school health services, but also to make a change of practice when doing so. We argue that it is important to have open and direct communication, as suggested by Brinley et al. (2024), even when there are not any concerns, to meet parents’ expectations, avoid unnecessary concerns, and gain the potential benefits of online screening tools. Lack of communication concerns from both parents and PHNs, may lead to missed opportunities for both identification of health and development challenges, as well as health promotion. As highlighted by Shonkoff et al. (2021), to facilitate a health-promotive dialogue, screening tools should be used to increase understanding for both providers and parents/children and create ground for common involvement and empowerment.

Conversely, when parents felt that the results were discussed and their concerns were addressed, their vulnerability was replaced with a sense of being seen and heard. They were then able to benefit from the positive outcomes, such as more information being available to the PHN, better structured conversations, and more time to address concerns. This helped to identify children with problems earlier and refer them for further evaluations, as noted previously (Hix-Small et al., 2007; Marks et al., 2009). Parents felt that addressing the specific screening results in the well-child visit was a benefit because it allowed them to talk with the PHN about the challenges they faced. The PHN’s guidance contributed to their increased awareness and helped the parents to become more confident in supporting the child’s development or facing the challenges. Even in the absence of developmental concerns, we argue that it is important to talk about the results with the parents because such conversations can provide an opportunity to educate parents about typical child development and the parent’s role in stimulating the child’s social–emotional, physical, and cognitive development (Shonkoff et al., 2021). However, it is important not only to rely on information from the screening tool but also to conduct thorough assessments during the well-child visit. The use of online screening tools should complement, not replace, the importance of listening to parents’ concerns (Valla et al., 2019).

Our results provide important information for understanding parents’ values and attitudes toward online screening tools, which are important for both the healthcare service and society at large. The results of this study might help well-child clinics and school health services adapt their practices to meet the family’s needs and expectations. The perspectives of parents provide important information about issues that influence whether and how they respond to such screening tools and how their use might create any unforeseen consequences that should be considered before national upscaling. In addition, exploration of the perspectives of parents who choose not to complete the online screening tools (those who were not included in this study) would be important for future studies aiming to obtain insights into the barriers preventing parents from completing online screening tools. Such exploration would also be useful for developing services to facilitate meeting all family needs.

Increasing health literacy and supporting parents to become confident and competent in their parenting roles are important and warranted (The Norwegian Directorate of Health, 2017). However, the parents in this study reported great variations in the PHNs’ handling of information, and this variation was strongly linked to parent satisfaction with the PHN’s handling of the information in the well-child visit. These variations should be explored in future studies to gain a better understanding of how and why these occur within services and how an online screening tool influences PHNs’ work.

The use of online screening tools can provide important information about the health, development, and well-being of children, thereby providing a basis for targeted interventions. The long-term outcomes of children who are followed using online screening tools should also be examined in future studies.

## Methodological Considerations

We applied thematic analysis, a well-recognized method for creating patterns of meaning in qualitative data (Braun & Clarke, 2022). To enhance the rigor of our study, we engaged multiple researchers in a reflexive and collaborative analytic process (Braun & Clarke, 2006), allowing for a richer and more nuanced interpretation of the data.

Our findings were strengthened by including a heterogeneous group of parents with children aged 0 to 7 years. Although a higher proportion of parents were mothers, five of the 16 parents interviewed were fathers, recognizing the importance of including their experiences. However, we did not explore gender-related influences on parent experiences in this study. Our study does not provide insights into non-responses’ experiences because we recruited only those participants who had completed the online screening tools before the well-child visit. Nevertheless, the rich material strengthens the trustworthiness of the findings which are transferable to similar initiatives for implementing online child health screening tools.

## Conclusion

The findings of this study indicate that although online screening tools may be accessible and usable, such tools pose both potential and pitfalls. By evoking novel insights about the child, the tool enhances parent engagement and understanding regarding their child’s health, development, and well-being both before and during well-child visits with the PHN. However, new expectations are raised. How the PHNs navigate these expectations influences whether the screening tool’s potential—such as more structured conversations with PHNs and earlier identification of issues, which can contribute to earlier interventions and improved outcomes for children—is harnessed or neglected. Notably, the study results highlight the importance of accurate information and open dialogue throughout the screening process to prevent evoking parental insecurity and vulnerability when responding to online screening tools.

## Supplemental Material

sj-docx-1-gqn-10.1177_23333936251342728 – Supplemental material for Parents’ Experiences with Online Screening Tools in Well-Child Clinics and School Health Services: A Qualitative Study Supplemental material, sj-docx-1-gqn-10.1177_23333936251342728 for Parents’ Experiences with Online Screening Tools in Well-Child Clinics and School Health Services: A Qualitative Study by Trine Holm, Elin Thygesen, Geir Inge Hausvik and Thomas Westergren in Global Qualitative Nursing Research
