# Corrigendum to “Parents’ Experiences with Online Screening Tools in Well-Child Clinics and School Health Services – A Qualitative Study”

**DOI:** 10.1177/23333936251388181

**Published:** 2025-10-14

Trine Holm, Elin Thygesen, Geir Inge Hausvik, Thomas Westergren, et al. Parents’ Experiences with Online Screening Tools in Well-Child Clinics and School Health Services – A Qualitative Study. *Global Qualitative Nursing Research*. Volume 12: 1–12, 2025. doi:10.1177/23333936251342728

In this article, the number of PHNs has been corrected from seven to eight in the “Participants and Recruitment” section. Additionally, the number of women has been corrected from seven to eight under the “PHNs observed (n = 8)” column in Table 1.

