# Supplementary material for: Parents’ Experiences with Online Screening Tools in Well-Child Clinics and School Health Services: A Qualitative Study
Source: Glob Qual Nurs Res. 2025 Jun 10;12:23333936251342728. doi: 10.1177/23333936251342728 (PMC12159474; doi:10.1177/23333936251342728)
Supplement: sj-docx-1-gqn-10.1177_23333936251342728 – Supplemental material for Parents’ Experiences with Online Screening Tools in Well-Child Clinics and School Health Services: A Qualitative Study [file sj-docx-1-gqn-10.1177_23333936251342728.docx]

## Interviews with Parents

Translated vertion

### Introductory Questions:

- How old are you?
- What is your role in relation to the child?
- What is your educational background?
- How old is your child?
- What is your child's gender?
- Which questionnaires did you answer?
- Tell us a bit more about your child. Who is he/she, and how is he/she doing?

### Main Questions about Answering the Questionnaire:

Who answered the online screening tool, and if you collaborate on this, how?

- Do you think similarly or differently about your child? Please elaborate...

How did you experience answering the questions?

- Scope
- Usability

How did you find the questions asked about your child? (Give the participant a copy of the questionnaires and ask them to read and think aloud)

- Relevant/Irrelevant?
- Positive/Negative tone?

How do you think your answers reflect your child’s health and well-being?

Would you say that the questions and your answers influenced how you think about your child, and if so, in what way?

### Now about the well-child visit with the public health nurse;

What experiences have you had previously with the well-child center/school health services and the public health nurse?

How did you experience this well-child visit?

How did you find your responses were used in the well-child visit between the public health nurse, your child, and you?

How did the responses influence the conversation between you, your child, and the public health nurse?

How do you think such an online screening tool can be used for the best possible follow-up of your family and your child?

### Final Comments:

- Do you have anything more to add?
- Thank you for participating
- May I contact you again if needed?
- Reminder about anonymity
